# Light-Adaptive Human Body Key Point Detection Algorithm Based on Multi-Source Information Fusion

**DOI:** 10.3390/s24103021

**Published:** 2024-05-10

**Authors:** Zhigang Hu, Chengwu Zhang, Xinzheng Wang, Aoru Ge

**Affiliations:** College of Medical Technology and Engineering, Henan University of Science and Technology, Luoyang 471023, China; hu.robert@163.com (Z.H.); 210321221630@stu.haust.edu.cn (C.Z.); 220320221769@stu.haust.edu.cn (A.G.)

**Keywords:** information fusion, lighting adaptation, key point detection

## Abstract

The identification of key points in the human body is vital for sports rehabilitation, medical diagnosis, human–computer interaction, and related fields. Currently, depth cameras provide more precise depth information on these crucial points. However, human motion can lead to variations in the positions of these key points. While the Mediapipe algorithm demonstrates effective anti-shake capabilities for these points, its accuracy can be easily affected by changes in lighting conditions. To address these challenges, this study proposes an illumination-adaptive algorithm for detecting human key points through the fusion of multi-source information. By integrating key point data from the depth camera and Mediapipe, an illumination change model is established to simulate environmental lighting variations. Subsequently, the fitting function of the relationship between lighting conditions and adaptive weights is solved to achieve lighting adaptation for human key point detection. Experimental verification and similarity analysis with benchmark data yielded R2 results of 0.96 and 0.93, and cosine similarity results of 0.92 and 0.90. With a threshold range of 8, the joint accuracy rates for the two rehabilitation actions were found to be 89% and 88%. The experimental results demonstrate the stability of the proposed method in detecting key points in the human body under changing illumination conditions, its anti-shake ability for human movement, and its high detection accuracy. This method shows promise for applications in human–computer interaction, sports rehabilitation, and virtual reality.

## 1. Introduction

Key point detection is crucial in the field of computer vision as it involves automatically identifying and locating specific body parts in images or videos [1], such as shoulders, elbows, wrists, knees, and ankles. This task has diverse applications in areas like sports rehabilitation [2,3], human–computer interaction [4], virtual reality [5,6], and security monitoring [7]. Precise identification and tracking of key points empower computers to understand and imitate human movements, postures, and actions more effectively [8,9], thereby creating valuable possibilities for a wide range of applications.

Initially, traditional machine learning methods were used to estimate human poses. For example, Shakhnarovich et al. used parameter-sensitive hash functions to estimate human joints [10]. With the rapid development of computer technology and depth sensing equipment, depth cameras based on machine learning algorithms are widely used to obtain key point information on the human body and play a role in various task scenarios. For example, Lu et al. used images collected by depth cameras to construct a data set and constructed an FBN (fast and lightweight deep learning network), focusing more on facial and hand details to achieve real-time three-dimensional human body reconstruction [11]. Azhar proposed an automatic gender classification system for real-time multi-view gait using depth cameras, which outperformed existing methods using minimal features and high accuracy [12]. With the rapid development of deep learning in the field of computer vision, a series of deep models for human body key point detection have also been released. Kim et al. used the skeletal posture estimated by the deep learning method Mediapipe as input and used the fast optimization method uDEAS (the univariate dynamic encoding algorithm for searches) to propose a 3D human posture estimation system to monitor whether elderly people living alone have fallen [13]. Estrada et al. used key human body points detected by Mediapipe to further identify correct and incorrect sitting postures while working from home [14]. In addition, other deep models have also achieved remarkable results in key point detection. For example, Byeon et al. proposed an integrated deep model that combines the majority method and the average method for gesture recognition. The results show that the InceptionResNetV2s integrated system using 5 preprocessing methods shows good performance [15]. InceptionResNetV2 is a combination of the Inception and ResNet series. It includes a backbone layer, three types of inception modules (A, B, and C), and two types of reduction modules (A and B). The backbone layer is responsible for receiving the input image and extracting the initial features. The three types of inception modules work to enhance the understanding of input images through multi-scale convolution. Reduction modules are designed to decrease the size of feature maps to improve computational efficiency.

The Kinect camera, which is primarily used for depth perception and motion tracking, has an RGB image component that does not perform as well as a specialized camera in low-light environments. Its image processing power may not be sufficient to cope with these complex lighting conditions. We hope to improve the accuracy and stability of human key point detection under changing lighting conditions through improvements at the software or algorithm levels. Inspired by previous research on human key point detection, this paper combines the Kinect depth camera and Mediapipe technology, and proposes a multi-source information fusion algorithm for light-adaptive human key point detection, which solves the jitter problem of Kinect camera in detecting key points, and improves the accuracy and stability of human key points under changing lighting conditions. The main contributions of this paper are as follows:

(1) Realizing multi-source information fusion, making full use of the human key point data obtained from depth image and RGB image detection, and improving the accuracy and stability of key point detection.

(2) Applying the L-BFGS optimization algorithm to find the fitting function of the relationship between light conditions and weights, and realizing the light adaption of human key point detection.

(3) Comparing and analyzing the effect of human key point detection between this paper’s method and Kinect and Mediapipe in gait balance training and center of gravity transfer training. The results show that the light-adaptive human key point detection algorithm proposed in this paper with multi-source information fusion is more accurate and stable.

## 2. Related Work

### 2.1. Kinect Human Body Key Point Detection Algorithm

Kinect 2.0 acquires scene depth point cloud data based on time-of-flight technology [16] and then maps these data to a two-dimensional plane to create a depth image [17]. Probabilistic prediction of body parts is carried out from deep contrast features of images via random decision forests. Formula (1) is used to obtain the learning probability P that the pixel x belongs to the body part c.

(1) $$P\left(c|I,x\right)=\frac{1}{T}\sum_{t=1}^{T}P_{t}\left(c|I,x\right)$$

where t represents the decision tree; T represents the number of decision trees; $P_{t}\left(c|I,x\right)$ represents the discrete probability distribution obtained after classifying pixels by the decision tree No. t; and I is the depth image acquired by the Kinect camera. To aggregate the learned probabilities for all pixels of the image, the depth estimate $f_{c}\left(\overset{\wedge }{x}\right)$ of the body part c is calculated using Formula (2).

(2) $$f_{c}\left(\overset{\wedge }{x}\right)\propto \sum_{i=1}^{N}\omega_{ic}\exp\left(-{\left\|\frac{\overset{\wedge }{x}-{\overset{\wedge }{x}}_{i}}{b_{c}}\right\|}^{2}\right)$$

where $\overset{\wedge }{x}$ is the coordinate of the three-dimensional world space; N is the number of pixels in the depth image; ${\overset{\wedge }{x}}_{i}$ is the coordinate of the pixel $x_{i}$ reprojected to the three-dimensional space according to its depth value; $b_{c}$ is the bandwidth of each body part learned. $\omega_{ic}$ represents the weighting coefficient of each pixel $x_{i}$ of image I.

However, when using the Kinect depth camera for dynamic human body key point detection, the detection results have a jitter problem. The reasons for these problems include the following: (1) When the human body moves quickly or takes complex postures, the depth image may be blurred due to human body movement [18,19], affecting the accuracy of key point detection. (2) Depth cameras utilize infrared light to capture depth information; however, the depth measurements may fluctuate as the human body moves, leading to potential failures in establishing connections between crucial points. Consequently, this may result in the detected position exhibiting jitter in space [20]. (3) When key points of the human body are occluded by other body parts or external objects, the depth camera may not be able to accurately identify the occluded key points, resulting in inaccurate detection results [21,22]. Although Kinect can provide good depth information, the accuracy of its human body key point detection may not be enough to support certain application scenarios [23], such as human body key point detection solutions that require higher accuracy in medical or scientific research.

### 2.2. Mediapipe Human Body Key Point Detection Method

The Mediapipe framework is based on the BlazePose algorithm proposed by Bazarevsky et al. [24], which is a lightweight, high-fidelity body pose-tracking network dedicated to human pose estimation on mobile devices. During the inference process, a Detector-Tracker design is adopted, which includes a lightweight human pose estimation detector and a pose-tracking network. The model uses a face detector to locate the pose region of interest (ROI) of the image and then passes it to the pose-tracking network to predict the coordinates of key points on the human body.

**Figure 1:**
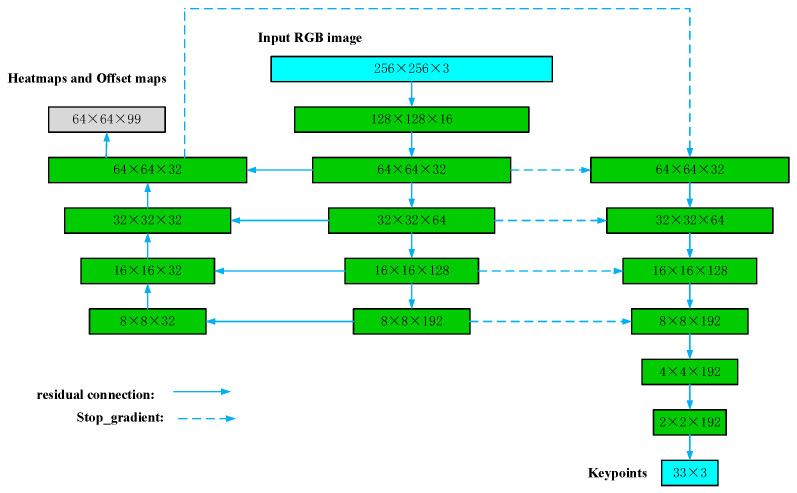
BlazePose neural network structure: fusion of heat maps and regression methods.

The BlazePose algorithm combines two mainstream methods based on heat maps and regression, as shown in Figure 1. Heatmaps and offset losses are used in the training phase to remove the corresponding output layer from the model before running inference, thereby reducing the complexity of the model and improving the lightweightness of the model. The use of image scaling and translation enhancement allows the tracker to handle the alignment of body movements and body distortions between frames. During the training process, occlusion data of simulated key points are added to predict invisible key points, which improves the robustness of the algorithm.

However, the BlazePose algorithm cannot correctly detect human body key points under poor lighting conditions. The main reason is that Mediapipe uses computer vision to analyze images, and it relies on specific features and brightness in RGB images to detect human body key points. When lighting conditions are poor, the contrast between the human body in the scene and the background is reduced, and the image captured by the camera is very blurry. The image lacks enough details and feature extraction becomes more difficult [19], which makes it difficult for the algorithm to accurately identify key points of the human body. Additionally, Mediapipe uses deep learning models to detect key points, and these models often rely on large-scale, diverse data sets during training. If there is a lack of images with poor lighting conditions in the training data, the generalization effect of the model may be affected [25].

## 3. Light-Adaptive Human Body Key Point Detection Algorithm Based on Multi-Source Information Fusion

To solve the above-mentioned problems in the human body key point detection by Kinect and Mediapipe, in this paper, combining the advantages of these algorithms in human key point detection, a multi-source information fusion light adaptive human key point detection algorithm is proposed. A light change model is first constructed to simulate the environmental light change. Then, the fitting function of the relationship between light conditions and adaptive weights is solved, and finally, the light adaptive human key point detection is realized. In this study, human limb joints are selected as data collection objects, including shoulder joints, elbow joints, wrist joints, hip joints, knee joints, and ankle joints. The corresponding relationship between Kinect and Mediapipe [26] joints is shown in Table 1.

### 3.1. Multi-Source Information Collection and Spatial Alignment

Multi-source information includes human body key point data U obtained by detecting RGB images by Mediapipe and human body key point data V obtained by detecting depth images by Kinect, as shown in Formula (3).

(3) $$\left\{\begin{array}{c} U=\left(X_{u},Y_{u},Z_{u}\right)\\ V=\left(X_{v},Y_{v},Z_{v}\right) \end{array}\right.$$

where X, Y, and Z represent the position of the key point data in the coordinate system.

To achieve data space alignment, this article uses the coordinate system in which U is located as the reference coordinate system, uses Zhang Zhengyou’s calibration method [27] to calibrate the Kinect camera, and solves its internal and external parameters. Through coordinate transformation, V is mapped from its coordinate system to the reference coordinate system to obtain data $V^{\prime }$. The coordinate transformation formula is as follows:

(4) $$s\left[\begin{array}{c} X_{v^{\prime }}\\ Y_{v^{\prime }}\\ Z_{v^{\prime }}\\ 1 \end{array}\right]=\left[\begin{array}{cccc} f_{x} & 0 & d_{x} & 0\\ 0 & f_{y} & d_{y} & 0\\ 0 & 0 & 1 & 0 \end{array}\right]\cdot \left[\begin{array}{cc} R & T\\ \vec{0} & 1 \end{array}\right]\cdot \left[\begin{array}{c} X_{u}\\ Y_{u}\\ Z_{u}\\ 1 \end{array}\right]$$

where $V^{\prime }$ is the result of V converted to the reference coordinate system; s represents the scale factor; $f_{x}$ and $f_{y}$ are the focal lengths of the depth camera; $d_{x}$ and $d_{y}$ are the optical center coordinates of the depth camera; R represents the camera rotation matrix; and T is the camera’s internal reference, representing the translation matrix.

### 3.2. Build a Lighting Change Model

To realize the goal of this paper’s algorithm to be adaptive to light changes, it is necessary to generate images under different light conditions. However, simply applying inverse gain to reduce the color channel intensity leads to a general loss of high-light information in the generated images. However, simply applying inverse gain to reduce color channel intensity results in a general loss of highlight information in the resulting image. Therefore, based on applying inverse gain to reduce the intensity of the color channel, this paper uses the function $f\left(x,g(J)\right)$ to save the image highlight to solve the problem [28]. The constructed model is as follows:

(5) $$\left\{\begin{array}{c} f\left(x,g(J)\right)=\max\left(\frac{x}{g(J)},\left(1-\alpha \left(x\right)\right)\left(\frac{x}{g(J)}\right)+\alpha \left(x\right)x\right)\\ \alpha \left(x\right)={\left(\frac{\max\left(x-t,0\right)}{1-t}\right)}^{2} \end{array}\right.$$

where x represents the set inverse gain; t represents the threshold; $g\left(J\right)$ is the value of the CIE-XYZ color space; and J is the value of the (R, G, B) channel in the sRGB color space. When g(J)≤1 or x≤t, the function is linear; when g(J)>1 and x>t, the function is a cubic transformation, which enhances the contrast of the image and makes the bright details in the image more prominent. Therefore, the $f\left(x,g(J)\right)$ function can retain more information about the highlighted part of the generated image.

After obtaining the linear value $f\left(x,g(J)\right)$ of the CIE-XYZ color space, gamma transformation is applied to convert the linear value to the sRGB color space, as shown in Formula (6), so that the image display is more consistent with the human eye’s perception of brightness. Mediapipe detects different illumination images generated by the illumination change model and obtains human body key point data $U^{\prime }$.

(6) $$C_{sRGB}=\left\{\begin{array}{cc} 12.92f\left(x,g(J)\right) & 0\leq f\left(x,g(J)\right)<0.0031\\ 1.055f{\left(x,g(J)\right)}^{1/2.4}-0.055 & 0.0031\leq f\left(x,g(J)\right)<1 \end{array}\right.$$

### 3.3. Multi-Source Key Point Information Fusion and Lighting Adaptation

This paper uses the key point data $D\left(X_{d},Y_{d},Z_{d}\right)$ collected by the motion capture system Vicon as the benchmark data [29], applies the L-BFGS optimization algorithm [30] to minimize the mean square error between the fused data L and the benchmark data, and calculates the optimal weight $h_{best}$, as shown in Formula (7).

(7) $$\left\{\begin{array}{l} L=h_{best}U^{\prime }+(1-h_{best})V^{\prime }\\ Mse\left(h\right)=\frac{1}{n}\sum_{i=1}^{n}{\left(L_{i}-D_{i}\right)}^{2} \end{array}\right.$$

The sigmoid function is used to fit the optimal weight $h_{best}$ to achieve illumination adaptation for human body key point detection. The algorithm described in Algorithm 1 is applied to minimize the mean square error of the estimated weight and the optimal weight, to calculate the optimal estimated parameters $a_{est}$ and $b_{est}$, and to substitute them into Formula (8) to obtain the adaptive fitting weight $h_{est}$ under different lighting conditions.

(8) $$h_{est}=\frac{1}{1+e^{-a_{est}\left(\bar{x}+b_{est}\right)}}$$

Then, under changing lighting conditions, the lighting adaptive data $L^{\prime }$ is as follows:

(9) $$L^{\prime }=h_{est}U^{\prime }+\left(1-h_{est}\right)V^{\prime }$$

where $L^{\prime }$ represents the final result of human key points after fusing multi-source information under simulated different lighting conditions, $U^{\prime }$ represents Mediapipe detecting simulated different lighting images to obtain human key point data, and $V^{\prime }$ represents the result of Kinect data after coordinate transformation. The above pseudocode for solving the optimal weight value is shown in Algorithm 1.
**Algorithm 1.** Optimal weight solution algorithm**Input**: Objective function Mse(h); Initial value $h_{0}$**Output**: Optimal solution $h_{best}$ **Abort condition:** gradient threshold $\varepsilon =1e^{-5}$
1: Select the initial point $h_{0}=0.5$; Store the latest num iteration data;2: k = 0, $H_{0}$ = I, $r=\nabla Mse(h_{0})$;3: if $\left\|\nabla Mse(h_{k+1})\right\|>\varepsilon$ then 4:  // Calculate the feasible direction for this iteration  $p_{k}=-r_{k}$
5:  // Calculate the step size $\alpha_{k}>0$ and perform a one-dimensional search on the following formula  $Mse(h_{k}+\alpha_{k}p_{k})=\min Mse(h_{k}+\alpha p_{k})$ 6:  // Update weights h   $h_{k+1}=h_{k}+\alpha_{k}p_{k}$ 7:  if k>num, Keep the most recent num vector pairs and delete them $\left(s_{k-num,}t_{k-num}\right)$ 8:  // Calculate and keep   $s_{k}=h_{k+1}-h_{k}$;$t_{k}=\nabla Mse(h_{k+1})-\nabla Mse(h_{k})$ 9:  // Use the two-loop recursion algorithm to find $r_{k}$   $r_{k}=B_{k}\nabla Mse(h_{k})$
10: k=k+1, and go to 311: Else12:  return $h_{best}$; 13: end if

The L-BFGS algorithm is a quasi-Newtonian method widely used for solving unconstrained nonlinear minimization problems. Its core idea is to approximate the optimal solution by estimating the inverse of the Hessian of the objective function. Compared to the traditional gradient descent method, L-BFGS typically exhibits a faster convergence rate in dealing with high-dimensional problems, making it suitable for large-scale problems. In our study, the L-BFGS algorithm is utilized to minimize the error function of multi-source key point information fusion and illumination adaptation. The error function encompasses the difference between multiple key information sources and the relevant terms of light adaptation. L-BFGS iteratively optimizes parameters to minimize errors and obtain more accurate pose estimation results. Its efficient processing of the high-dimensional parameter space and relatively small storage requirements make it the chosen algorithm for this study. In our experiments, L-BFGS demonstrated commendable performance in reducing critical information fusion errors and adapting to different lighting conditions. Through the use of the L-BFGS optimization algorithm, the model’s parameters can be adjusted more effectively, leading to improved accuracy of multi-source information fusion and enhanced adaptability to different lighting conditions.

### 3.4. Human Body Key Point Estimation

Due to the influence of illumination changes and human movement, the adaptive illumination data $L^{\prime }$ contain random noise and motion noise. To smooth the fused data and obtain the optimal estimate of human body key points, this paper uses the Kalman filter algorithm [31] for state estimation. The prediction stage utilizes data from the previous 30 frames to analyze and estimate the potential position of the key point in the next second. The state prediction formula is as follows:

(10) $$\overset{\wedge }{L^{\prime }}\left(k\right)=AL^{\prime }\left(k-1\right)$$

where $L^{\prime }$ represents the estimated value at time k−1; $L^{\prime }\left(k\right)$ represents the predicted value at time k; and A is the state transition matrix. The covariance prediction formula is as follows:

(11) $$\overset{\wedge }{P}\left(k\right)=AP\left(k-1\right)A^{T}+Q\left(k\right)$$

where $P\left(k-1\right)$ represents the a priori estimated covariance at time k−1; $P\left(k\right)$ represents the predicted covariance at time k; and $Q\left(k\right)$ represents the process noise covariance set to 0.08I.

In the update stage, the estimated value in the prediction stage is corrected based on the Kalman gain to obtain the corrected optimal estimated value. The state estimate update formula is as follows:

(12) $$L^{\prime }\left(k\right)=\overset{\wedge }{L^{\prime }}\left(k\right)+K\left(k\right)\left[z\left(k\right)-H\overset{\wedge }{L^{\prime }}\left(k\right)\right]$$

where $L^{\prime }\left(k\right)$ represents the optimal estimated value at the time k; $z\left(k\right)$ represents the measured value; and H represents the observation matrix. $K\left(k\right)$ represents the Kalman gain and the calculation formula is as follows:

(13) $$K\left(k\right)=\frac{\overset{\wedge }{P}\left(k\right)H^{T}}{H\overset{\wedge }{P}\left(k\right)H^{T}+R\left(k\right)}$$

where $R\left(k\right)$ represents the observation noise covariance matrix at k time. Because the position transformation of human body key points has uncertainty in three directions, the R matrix is set to 0.2I.

The process described above effectively minimizes interference from human motion and sensors, which helps enhance the precision and consistency of the data. Simultaneously, the gathered data can be continuously updated and processed in real-time using dynamic forecasting, ensuring the real-time aspect of data transmission.

## 4. Results

This section analyzes the detection effect of the key point detection algorithm, Kinect, and Mediapipe algorithm in the field of rehabilitation through experiments. Two kinds of lower limb rehabilitation exercises are designed, namely gait balance function rehabilitation training and center of gravity transfer rehabilitation training. In the experiment of key point jitter, the key point detection effect of Kinect and the proposed algorithm in the process of human motion is compared. In the light adaptive experiment, the key point detection effect of Mediapipe, OpenPose, HRNet, and the proposed algorithm is compared under different lighting conditions. In the comprehensive experiment, the overall detection effect of Kinect, Mediapipe, OpenPose, HRNet, and this method is compared.

This article mainly uses MSE (mean square error), R2 (coefficient of determination), cosine similarity, difference percentage, joint accuracy, and CV (coefficient of variation) as the evaluation criteria for key point data. MSE measures the average of the squared error between the predicted value and the actual value. In the key point data evaluation, the difference between the predicted value and the actual value is calculated for each key point location, and the difference is squared and averaged. The smaller the MSE, the more accurate the model is in predicting the key point location. In the literature [32], MSE is used to determine the constant value that minimizes the error and how close the estimate or prediction is to the actual data. R2 measures how well the forecast data match the baseline data. In the key point data evaluation, R2 measures how well the model fits change in the key point location. R2 is calculated in a way that takes into account the ratio of the variance predicted by the model to the variance of the actual observed values, with values ranging from 0 to 1. The closer to 1, the better the fitting effect, and the closer to 0, the worse the fitting effect [33]. Cosine similarity measures the cosine of the angle between two vectors. In the evaluation of key point data, cosine similarity is used to measure the directional consistency between the predicted key point location and the actual location. Its value is between −1 and 1, and the closer it is to 1, the more consistent the direction is. Reference [34] introduced cosine similarity to measure the distance between postures as an indicator to assess the quality of rehabilitation movements. Percentage difference is a measure used to quantify the relative difference between two pieces of data. In key point data evaluation, the percentage difference measures the relative error between the predicted key point location and the actual location. It takes into account the percentage of relative differences, making it applicable to data at different scales [35]. Joint accuracy is the ratio between the number of correctly predicted key points and the total number of key points in the key point detection task. It represents the consistency of the output joint position with the actual joint position within a given threshold range. The study in [36] takes joint accuracy as an index to evaluate the performance of fall detection algorithms based on node characteristics After referring to related work on threshold setting in the literature [37,38], the pixel values 8 and 6 are selected as the thresholds for measuring the joint accuracy in this paper. The coefficient of variation (CV) is a measure of the relative stability of data, which can provide useful information about the volatility of data. The reference standard for the coefficient of variation is as follows: with less than 10%, low variability, relatively stable data; from 10% to 20%, medium variability, moderate fluctuation of data; with more than 20%, high variability, large fluctuation of data, relatively unstable. The Structural Similarity Index (SSIM) [39] and Peak Signal-to-Noise Ratio (PSNR) [40] were used as metrics to assess the similarity between the generated simulated and real-light images. SSIM is a measure of the visual similarity of two images, which reflects the degree of similarity between the two images in terms of structural information, brightness and contrast. SSIM is a measure of visual similarity between two images that reflects the degree of similarity in terms of structural information, brightness, and contrast, and is more reflective of the human visual system’s perception of image quality than the traditional pixel-level error metrics. SSIM ranges from −1 to 1. A value of 1 means that the two images are exactly the same; 0 means that there is no similarity whatsoever between them; and a negative value is rare and usually indicates that there is an anomaly in the comparison results. An SSIM greater than 0.85 is considered to be a good similarity between the two images, which is suitable for most commercial and practical applications. An SSIM greater than 0.85 is considered good similarity between images and is suitable for most commercial and practical applications. Peak Signal-to-Noise Ratio (PSNR) is a widely used metric for evaluating the fidelity of images, with a theoretical range of values from 0 to infinity, measured in decibels (dB), where higher values indicate better fidelity, and where PSNR in the range of 30 dB to 40 dB can be considered to provide good fidelity between images. The above indicators are specified in the following formula:

(14) $$MSE(F,D)=\frac{1}{n}\sum_{i=1}^{n}{\left(F_{i}-D_{i}\right)}^{2}$$

(15) $$Similarity(F,D)=\frac{\sum_{i=1}^{n}\left(F_{i}\times D_{i}\right)}{\sqrt{\sum_{i=1}^{n}F_{i}^{2}}\times \sqrt{\sum_{i=1}^{n}D_{i}^{2}}}$$

(16) $$R^{2}=1-\frac{SS_{res}}{SS_{tot}}$$

(17) $$\mathrm{Percentage}(F,D)=\frac{1}{n}\sum_{i=1}^{n}\left|\frac{F_{i}-D_{i}}{\frac{1}{2}(F_{i}+D_{i})}\right|\times 100\%$$

(18) $$JointAccuracy=\frac{num_{Correct}}{num_{Total}}$$

(19) $$CV=\frac{\sigma }{\bar{m}}$$

(20) $$SSIM(p,q)=\left(\frac{2\mu_{p}\mu_{q}+C_{1}}{\mu_{p}^{2}+\mu_{q}^{2}+C_{1}}\right)\times \left(\frac{2\sigma_{pq}+C_{2}}{\sigma_{p}^{2}+\sigma_{q}^{2}+C_{2}}\right)$$

(21) $$PSNR(p,q)=10\cdot \log_{10}\left(\frac{MAX_{p}^{2}}{MSE(p,q)}\right)$$

where F represents the key point data output by each method; $SS_{res}$ represents the sum of squares of the residuals, and $SS_{tot}$ is the sum of squares of the difference between the data and the benchmark data, which represents the total sum of squares, that is, the difference between the average value of the data and the benchmark data sum of the square. $num_{Correct}$ represents the exact number of joints within the threshold range, and $num_{Total}$ represents the total number of joints. σ represents the standard deviation of the mean square error of the repeated series and $\bar{m}$ represents the mean of the mean square error of the repeated series. p,q represent the real illumination image and the simulated illumination image, respectively. $\mu_{p}$,$\mu_{q}$ represent the average luminance of the images p and q, respectively. $\sigma_{p}^{2}$,$\sigma_{q}^{2}$ represent the variance of images p and q, respectively. $\sigma_{pq}$ is the covariance of images p and q. $C_{1}$ and $C_{2}$ are the small constants that are introduced in order to stabilize the denominators. $MAX_{p}$ is the image’s maximum pixel value.

The experimental environment is Intel(R) Core(TM) i9-12900H @ 2.50 GHz CPU, NVIDIA Geforce RTX 3060 GPU (Lenovo Group, Beijing, China), and 16 GB running memory. The operating system is Windows 10, Vicon Motion Capture System, Infrared Reflective Marker Sphere (Vicon Motion Systems Ltd., Oxford, UK), and Kinect 2.0 camera (Microsoft Corporation, Redmond, WA, USA). The Kinect 2.0 camera is about 1.5 m from the ground and 1.6–2.2 m from the tester. It is placed horizontally towards the tester.

**Figure 2:**
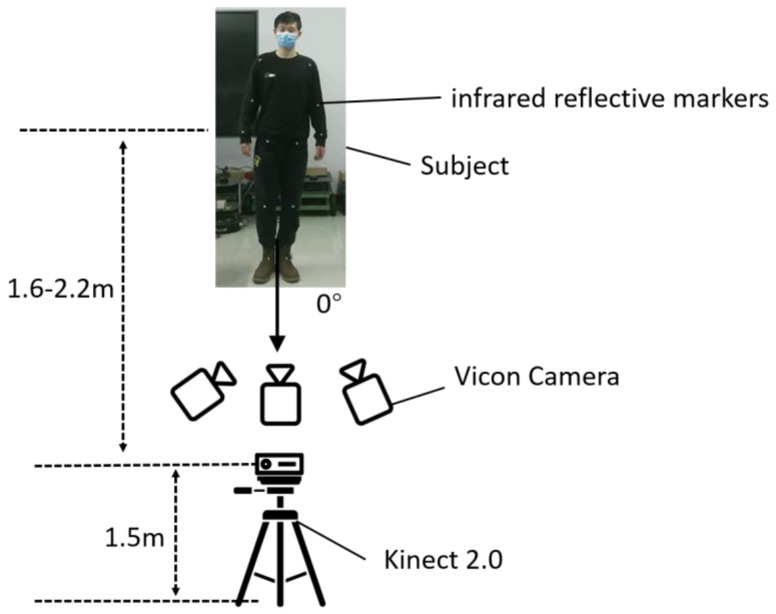
Schematic diagram of data acquisition.

A total of 13 participants’ joint point data were collected in this experiment, and the subjects stood facing the Kinect camera head-on. The three cameras of the Vicon system were located on the upper left, upper front, and upper right of the participants, and there was no occlusion of the participants by other objects. Under the guidance of a professional doctor, we attached infrared reflective marker spheres with a diameter of 14 mm to the limb joints of the subjects, including the shoulder, elbow, wrist, hip, knee, and ankle joints at a total of 12 joints, and the reflective marker points were as close as possible to the centers of the anatomical joints, as shown in the schematic in Figure 2. 

Experiments were conducted in an indoor environment to ensure good lighting conditions, avoiding reflective surfaces and other factors that could affect the performance of the Kinect camera and Vicon system. The Vicon system was calibrated using the Vicon Nexus software (Software version number: Vicon Nexus 2.8.2) and a calibration cross. The Kinect camera was calibrated using a calibration plate. The coordinates of the Vicon marker points were converted to the Kinect coordinate system via a coordinate transformation and then transferred to the 2D image coordinate system via coordinate mapping. Before and after each data acquisition, participants were asked to assume a specific “T” pose as a signal for the beginning and end of the session. Each participant followed the two rehabilitation maneuvers in the text for data acquisition, and each rehabilitation maneuver was repeated five times with the same sequence, for a total of 2 × 13 × 5 = 130 maneuver sequences, and the coordinates of joints captured by the Vicon system and the Kinect camera and the RGB video were recorded. Because the frequency of the Vicon system is 100 Hz and the frequency of the Kinect camera is 30 Hz, to ensure the time synchronization of the two devices, the data captured by the Vicon were downsampled and the frequency synchronized to 30 Hz. When the data were recorded, the timestamps of the data were synchronized to facilitate the time calibration. After time calibration, each frame of data acquired by the Vicon system and the Kinect camera contained the 3D coordinates of 12 joint points of the human body. The number of frames per data sequence ranged from 400 to 1200 frames, depending on the different movement speeds of each participant.

### 4.1. Rehabilitation Action Design

**Figure 3:**
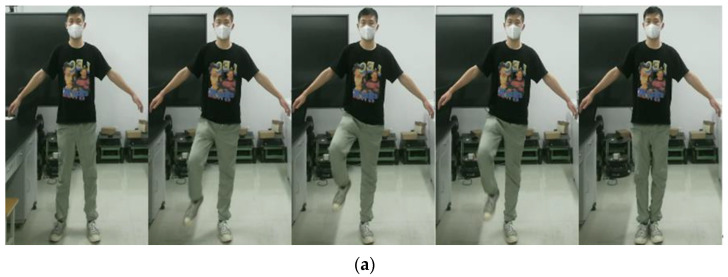

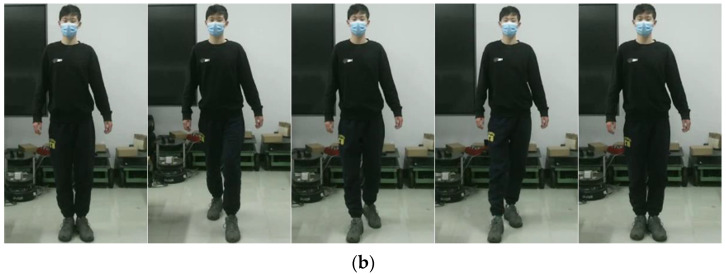
Rehabilitation action design. (**a**) Represents the gait balance function rehabilitation action. (**b**) Represents weight-shifting rehabilitation movements.

During the gait balance function rehabilitation training, the participant initially assumes a standing posture. They must maintain a stable upper body and then elevate the upper limbs sideways from the body. The lower limbs are gently raised and lowered, lifting the legs as high as possible and walking in a straight line to one side. This action is illustrated in Figure 3a. For weight transfer rehabilitation training, the participant first stands in position. They then support one leg and maintain its position while the other leg takes a small step forward, transferring the body’s weight to this leg. Next, they perform a lunge with bent knees, shifting their weight onto the supporting leg. Subsequently, they retract the other leg and take a small step back, transferring the body’s center of gravity from the supporting leg to this leg. This cycle is repeated three times, as demonstrated in Figure 3b.

### 4.2. Key Point Jitter Experiment

**Figure 4:**
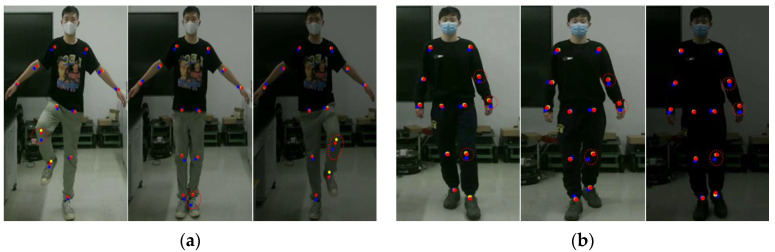
Key point jitter experiment. (**a**) Represents the results of gait balance function rehabilitation training. (**b**) Represents the results of weight-shifting rehabilitation training.

The testers carried out gait balance function rehabilitation and center of gravity transfer rehabilitation training according to the movement essentials and intercepted three frames of images during the rehabilitation training process. The key point detection results of Kinect and this method are shown in Figure 4.

In Figure 4, the red dots represent the key point data visualization results obtained by the method, the blue dots represent the key point data obtained by Kinect, and the yellow dots represent the key point baseline data. These two rehabilitation movements are designed to exercise the body’s balance and coordination abilities, and both emphasize the coordinated movements of the hip, knee, and ankle joints. As can be seen from the above experimental results, the knee and ankle joint positions detected by Kinect have obvious jitters during movement, and the key point detection results deviate from the benchmark data, while the detection results of this method are more stable during human movement, and no joints occur. There is a small jitter problem. Because the lower limb joints are more obviously involved in the rehabilitation exercise process, the right knee joint was selected as a representative to draw the key point position curve.

**Figure 5:**
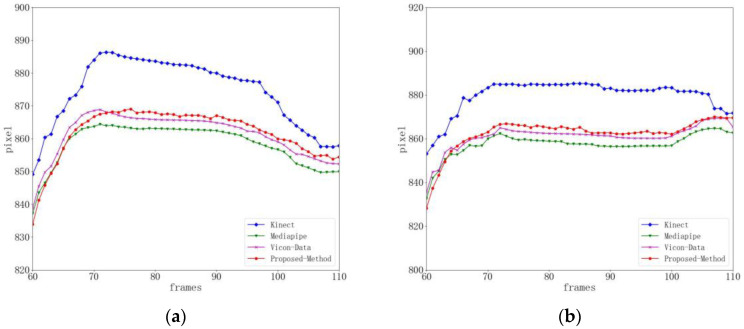
Right knee joint position curve during gait balance function rehabilitation training (key point jitter experiment). (**a**) Represents the x-position curve. (**b**) Represents the y-position curve.

Figure 5 shows the human body undergoing gait balance function rehabilitation training. Due to the tester’s right lower limb lifting and lowering, the right knee joint point data obtained by Kinect have noise and jitter, and the difference percentage from the baseline data is 9.32%. The detection results of Mediapipe and the method in this paper are more stable, with a percentage difference of 6.58% between Mediapipe and the baseline data, and a percentage difference of 7.24% between the method in this paper and the baseline data. When the threshold range is set to eight pixels, the joint accuracy of Kinect is 77.4%, the joint accuracy of Mediapipe is 85.5%, and the joint accuracy of this method is 90.8%, as shown in Table 2.

**Figure 6:**
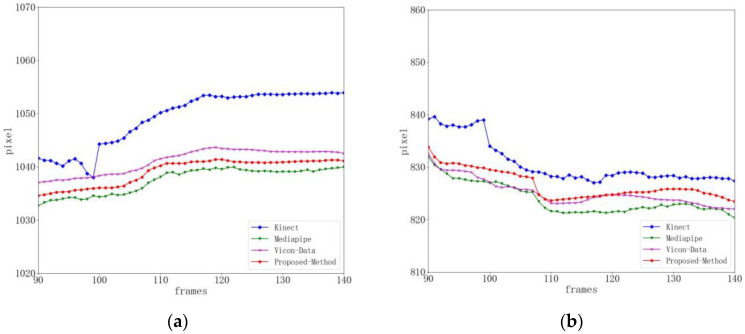
Right knee joint position curve during weight transfer rehabilitation training (key point jitter experiment). (**a**) Represents the x-position curve. (**b**) Represents the y-position curve.

It can be seen from Figure 6 that during the center of gravity shift rehabilitation training, because the movement range of the center of gravity shift rehabilitation training is small, the degree of jitter in the right knee joint position is smaller than that in the gait balance rehabilitation training. The difference percentage between Kinect and the benchmark data is 8.13%, the percentage difference between Mediapipe and the benchmark data is 5.99%, and the difference percentage between this method and the benchmark data is 6.54%. Compared with the Kinect method, the detection results of this method are more accurate and stable. When the threshold range is set to eight pixels, the joint accuracy of Kinect is 83.5%, the joint accuracy of Mediapipe is 87.6%, and the joint accuracy of this method is 91.9%, as shown in Table 3.

### 4.3. Lighting Adaptation Experiment

**Figure 7:**
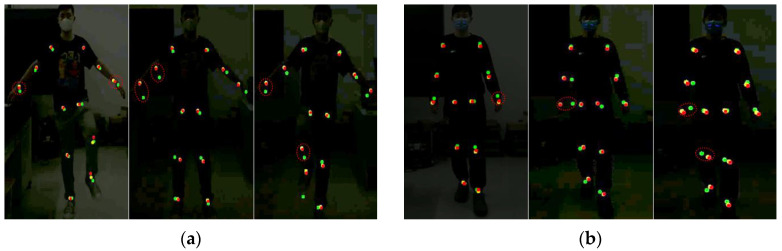
Light adaptation experiment. (**a**) Represents the results of gait balance function rehabilitation training. (**b**) Represents the results of weight-shifting rehabilitation training.

The illumination change model generates images with different illumination to simulate changes in environmental illumination. Three frames of images with different illumination brightness during the rehabilitation training process are intercepted. When the illumination changes, the key point detection results of Mediapipe and this method are shown in Figure 7.

In Figure 7, the red dots represent the key point data obtained by this method, the green dots represent the key point data obtained by Mediapipe, and the yellow dots represent the key point baseline data. It can be seen from the above experimental results that when the lighting conditions change, the Mediapipe method cannot correctly detect key points of the human body, especially areas with low discrimination such as wrist joints, knee joints, and ankle joints. There is a large discrepancy between the detection results and the benchmark data. The detection results of this method can accurately detect key points of the human body under different lighting conditions, and the detection results are more stable.

**Figure 8:**
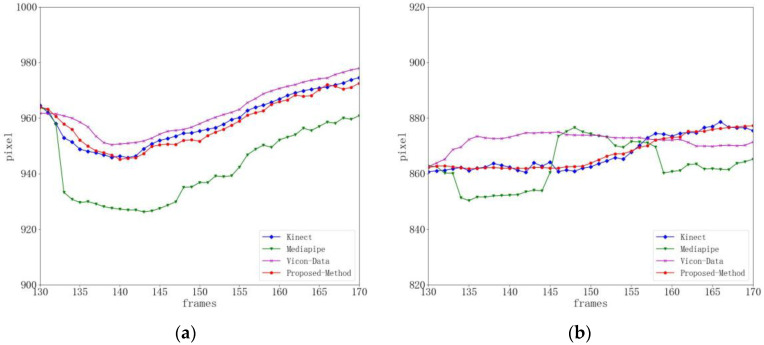
Right knee joint position curve during gait balance function rehabilitation training (lighting adaptation experiment). (**a**) Represents the x-position curve. (**b**) Represents the y-position curve.

As can be seen from Figure 8, when illumination changes, the right knee joint position obtained by the Mediapipe algorithm has a large deviation from the benchmark data, and the detection accuracy is low. This method is more accurate in the detection of the right knee joint position. By comparing the proposed method with the Mediapipe, OpenPose, and HRNet human key point detection algorithms, the results show that the percentage difference between Mediapipe and the baseline data is 15.34%, the percentage difference between Kinect and the baseline data is 8.93%, and the percentage difference between OpenPose and the baseline data is 12.58%. The percentage difference between the HRNet baseline data is 10.47%, and the percentage difference between the method and the baseline data is 8.84%. When the threshold range is set to eight pixels, the joint accuracy of Mediapipe is 59%, the joint accuracy of Kinect is 81%, the joint accuracy of HRNet is 76%, and the joint accuracy of the method is 84%, as shown in Table 4.

**Figure 9:**
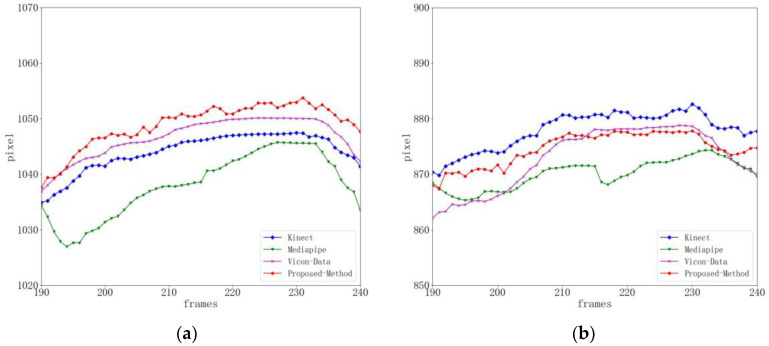
Right knee joint position curve during weight transfer rehabilitation training (lighting adaptation experiment). (**a**) Represents the x-position curve. (**b**) Represents the y-position curve.

As can be seen from Figure 9, in the process of the center of gravity transfer rehabilitation training, the right knee joint position obtained by the Mediapipe algorithm fluctuates greatly under the condition of light change, and there is a big deviation from the benchmark data. This method is more stable and accurate in detecting the right knee joint position. The percentage difference between Mediapipe and the baseline data is 14.92%, that between Kinect and the baseline data is 8.71%, that between OpenPose and the baseline data is 11.21%, that between HRNet and the baseline data is 9.67%, and that between the method and the baseline data is 7.94%. When the threshold range is set to eight pixels, the joint accuracy of Mediapipe is 72%, that of Kinect is 88%, that of OpenPose is 75%, that of HRNet is 79%, and that of the method is 90%, as shown in Table 5.

**Figure 10:**
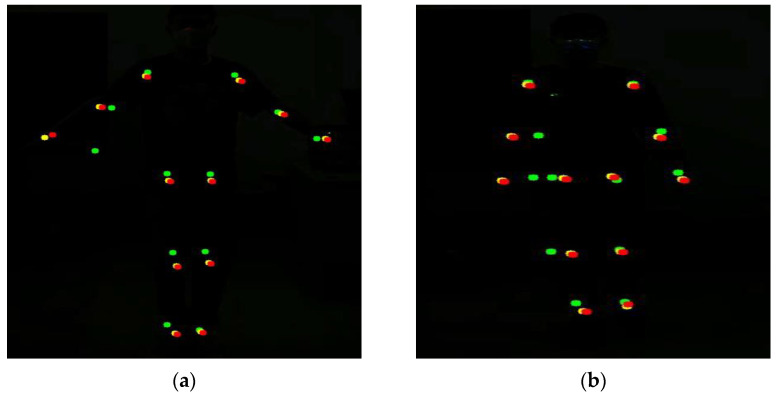
Experiments under actual bad lighting. (**a**) Results of rehabilitation training for gait balance function. (**b**) Rehabilitation training results of the center of gravity transfer.

To test the applicability of the algorithm proposed in this paper under real-light conditions, we conducted relevant experiments in the gait balance training and center of gravity transfer training tasks, as shown in Figure 10.

As can be seen from the above figure, under actual poor lighting conditions, a large deviation occurs between the key points of the human body detected by the Mediapipe method and the baseline data, which is due to the fact that the Mediapipe method is not able to accurately recognize the contours and features of the human body in the image under low light conditions. Low-light environments increase image noise and blurriness, resulting in a lack of image details, making it potentially difficult for Mediapipe’s vision algorithm to accurately detect human key points. In the gait balance training task, the percentage difference between Mediapipe and the benchmark data is 13.54% and the joint accuracy is 65%. The percentage difference between this paper’s method and the benchmark data is 7.35%, and the joint accuracy is 88%, as shown in Table 6. In the center of gravity shift training task, the percentage difference between Mediapipe and the benchmark data is 12.86% and the joint accuracy is 75%. The percentage difference between the method of this paper and the benchmark data is 6.83% and the joint accuracy is 91%, as shown in Table 7.

### 4.4. Comprehensive Comparative Experiment

In this experiment, three frames of images during the entire rehabilitation training process were intercepted, and the key point detection results of this method, the Kinect, and Mediapipe algorithms were comprehensively compared.

**Figure 11:**
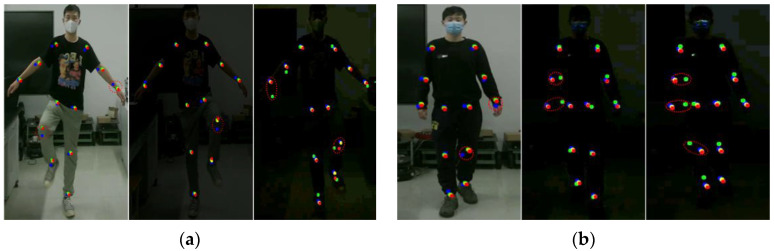
Comprehensive comparison of various methods. (**a**) Represents the results of gait balance function rehabilitation training. (**b**) Represents the results of weight-shifting rehabilitation training.

As can be seen from Figure 11, there are varying degrees of deviation between the human body key point detection results of Kinect and Mediapipe and the baseline data. Due to human movement, the Kinect detection results of knee and ankle joints are jittered; the Mediapipe method is sensitive to light, and under poor lighting conditions, its key point detection results will deviate significantly, especially in areas with low discrimination of the wrist joint. The overall detection effect of this method is more stable under changes in human movement and attention.

In addition, during the gait balance function rehabilitation and weight transfer rehabilitation training, this article collected key point data from thirteen testers. The average results of comparing the key point data obtained by each method with the benchmark data are shown in the Table 8 and Table 9, in which the threshold range for calculating joint accuracy is set to eight pixels.

The accuracy of the method in gait balance function rehabilitation training is 89%, and the accuracy in weight transfer rehabilitation training is 88%. As can be seen from the four objective evaluation indicators in the table, the key point data obtained by this method are closer to the benchmark data than that obtained by Kinect, Mediapipe, Openpose, and HRNet methods, and the detection results are more accurate.

To test the stability of the method of this paper in the repeated sequences of subjects, we performed a statistical analysis of the coefficient of variation (CV) of the mean square error obtained from the repeated sequences of two rehabilitation maneuvers for each subject, and the results of the analysis are shown in the table below.

The results in Table 10 are expressed as percentages, and the coefficients of variation of the mean square error for all 13 subjects with 5 repetitions of the sequence were lower than 10%. This indicates that the mean square error results are relatively stable with low variability in the subjects’ repeated sequences, which further validates the reliability and stability of our proposed method. In addition, we performed an analysis of variance (ANOVA) on the mean square error results of this paper’s method and the two base algorithms, and the specific results can be found in Table 11. The *p*-values of the ANOVA of the mean square error are lower than the significance level (α = 0.05) in the two movements we designed, namely, gait balancing and center of gravity transfer. This means that there is a significant difference between the mean values of this paper’s method and the two base algorithms, which indicates that the algorithm proposed in this paper produces a statistically significant improvement.

The key point data obtained by this method, before and after state estimation, and the comparison results with the benchmark data are shown in the figure below.

**Figure 12:**
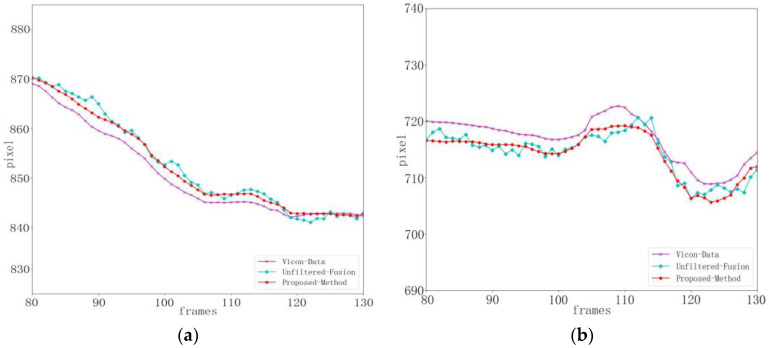
Right knee position and state estimation during gait balance function rehabilitation training. (**a**) x-position state estimation. (**b**) y-position state estimation.

**Figure 13:**
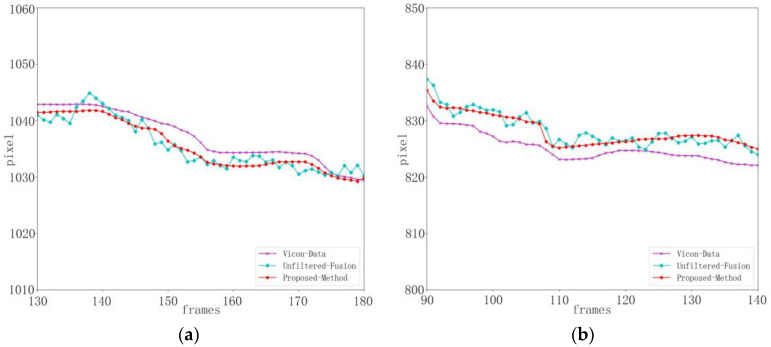
Estimation of right knee position and state during weight transfer rehabilitation training. (**a**) x-position state estimation. (**b**) y-position state estimation.

Figure 12 shows the state estimation results in the x and y directions of the right knee joint during gait balance function rehabilitation training. Figure 13 shows the state estimation results in the x and y directions of the right knee joint during the center of gravity transfer rehabilitation training. The cyan curve represents the result without state estimation by our method, the red curve represents the result after the state estimation of the key point location by our method, and the purple curve represents the baseline data. It can be observed from the figure that there is some movement and random noise in the fused data, which is mainly due to the dynamic changes and interference that may exist in the actual rehabilitation training. However, through the processing of critical point state estimation, we successfully attenuated the effect of these noises. The red curve is smoother than the cyan curve, indicating that the introduction of state estimation improves the accuracy and stability of data to a certain extent. This indicates that our method improves the reliability of data by reducing the effect of noise when processing data during rehabilitation training. In summary, by introducing state estimation, our method has shown some inhibition effects on motion and noise in experiments. Although there are some dynamic changes and interference, the processing of key point state estimation makes the data smoother and provides a more accurate and stable posture estimation for rehabilitation training. This is important for exercise monitoring and analysis in practical rehabilitation applications.

### 4.5. Comparison of Simulated and Real Lighting

We selected real-light images and simulated-light images in gait balance function rehabilitation training and center of gravity transfer rehabilitation training, respectively, and compared the differences in their histograms.

**Figure 14:**
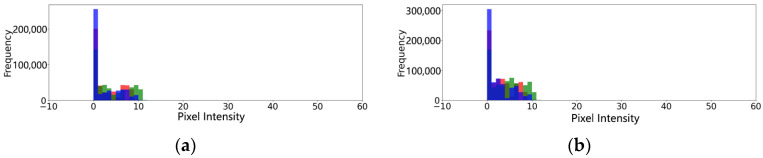
Light verification experiment of gait balance function rehabilitation training. (**a**) Histogram of real-light conditions. (**b**) Histogram of simulated-light conditions.

**Figure 15:**
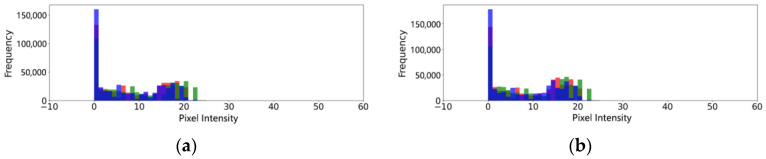
Light verification experiment of center of gravity transfer rehabilitation training. (**a**) Histogram of real-light conditions. (**b**) Histogram of simulated-light conditions.

As shown in Figure 14 and Figure 15, the histograms of simulated-light images and real-light images showed good agreement in the location and width of the main peaks in the gait balance rehabilitation training and the center of gravity transfer rehabilitation training. The red bars in the figure, show the intensity distribution of all pixels in the image on the red channel. The green bar, shows the intensity distribution of all pixels in the image on the green channel. The blue bars, show the intensity distribution of all pixels in the image on the blue channel. The other colors present on the graph are due to color mixing when the red, green, and blue bars overlap. We also used two metrics, ssim and psnr, to quantitatively assess the similarity between the generated simulated-light images and the real-light images. The results are shown in Table 12.

As shown in Table 12, during gait balance training, the SSIM and PSNR between the generated images and real images are 0.81 and 30.84, respectively. Although the SSIM is below the general standard, the overall similarity between the images is satisfactory when considering the PSNR results. In the center of gravity transfer training, the SSIM and PSNR between the generated and real images are 0.88 and 35.21, respectively, indicating good similarity between the images. Overall, the illumination transformation model effectively simulates real lighting effects.

## 5. Discussion

In this paper, a multi-source information fusion light adaptive human key point detection algorithm is proposed. It has the advantages of high detection accuracy and stable detection effect. In the key point jitter experiment, the method in this paper obtains 90.8% and 91.9% joint accuracy on the two designed training maneuvers, which solves the problem of joint point jitter to a certain extent. In the light adaptive experiment, the method in this paper obtains 84% and 90% joint accuracy on the two designed training actions, which solves the effect of environmental light changes on key point detection to a certain extent. In the comprehensive experiment, the method of this paper obtained 89% and 88% joint accuracy on the two designed training maneuvers, respectively. The overall performance of the algorithm is better than the comparative methods listed in the paper. It can be applied to the fields of sports rehabilitation, human–computer interaction, and virtual reality. For example, in the field of sports rehabilitation, more comprehensive and accurate data on human key points can be obtained, which help to monitor the posture and movement of patients in a finer way. This is important in the field of sports rehabilitation because in some cases, especially in joint movement recovery, more detailed information is needed to ensure that the patient is adopting the correct posture and movement. The method in this paper can be better adapted to rehabilitation scenarios in different environments and improve the robustness and reliability of the system. In the field of human–computer interaction (HCI), the combination of Kinect and Mediapipe technologies allows for finer and more natural interaction control. In HCI applications, this will lead to a higher level of user experience, where users can interact with the system more naturally, enabling more precise postural control and thus improved interactivity. At the same time, changes in light may pose challenges to gesture recognition and interactivity. Fusing data from multiple sources can provide more reliable gesture recognition while overcoming the problem of changing light. This has practical implications for HCI applications in different outdoor and indoor lighting conditions. In the field of virtual reality, the method in this paper can improve the realism and immersion of people in virtual environments. By capturing the user’s posture more accurately, the system can render virtual scenes more naturally and provide a more realistic virtual experience.

Although the multi-source information fusion algorithm proposed in this study shows effective results in dealing with different lighting conditions and motion jitter, there are still some limitations that need to be addressed for further improvement. One of the main limitations is that the accuracy of key point detection decreases in the case of drastic lighting changes or self-occlusion of joints. In addition, the performance of the method in dynamic environments with fast motion or complex backgrounds has not been extensively tested, which may pose a challenge in these common real-world application scenarios. To enhance the robustness and generalizability of the proposed algorithm, future work could focus on considering the incorporation of additional sensor modalities, improving the light adaptation model to handle a wider range of lighting conditions, and refining the algorithm to better handle occlusions and fast motion. In addition, integrating machine learning techniques to enable real-time adaptation and learning from new environments to further improve the performance and applicability of the system should be considered.

## Figures and Tables

**Table 1:** Correspondence between Kinect and Mediapipe joints.

Joint Name	Kinect Joint Index	Mediapipe Joint Index
Left Shoulder	4	11
Left Elbow	5	13
Left Wrist	6	15
Right Shoulder	8	12
Right Elbow	9	14
Right Wrist	10	16
Left Hip	12	23
Left Knee	13	25
Left Ankle	14	27
Right Hip	16	24
Right Knee	17	26
Right Ankle	18	28

**Table 2:** Key point detection results of right knee by Kinect and our method in gait balance rehabilitation training.

Method	Joint Accuracy (Threshold = 6)	Joint Accuracy (Threshold = 8)	Difference Percentage
Kinect	72.4%	77.4%	9.32%
Mediapipe	77.2%	85.5%	6.58%
ours	82.6%	90.8%	7.24%

**Table 3:** Key point detection results of right knee by Kinect and our method in weight transfer rehabilitation training.

Method	Joint Accuracy (Threshold = 6)	Joint Accuracy (Threshold = 8)	Difference Percentage
Kinect	74.8%	83.5%	8.13%
Mediapipe	78.3%	87.6%	5.99%
ours	83.2%	91.9%	6.54%

**Table 4:** Key point detection results of right knee by Mediapipe, Openpose, HRNet, and our method.

Method	Joint Accuracy (Threshold = 6)	Joint Accuracy (Threshold = 8)	Difference Percentage
Mediapipe	54%	59%	15.34%
Kinect	79%	81%	8.93%
OpenPose	62%	66%	12.58%
HRNet	72%	76%	10.47%
ours	80%	84%	8.84%

**Table 5:** Key point detection results of right knee by Mediapipe and our method.

Method	Joint Accuracy (Threshold = 6)	Joint Accuracy (Threshold = 8)	Difference Percentage
Mediapipe	61%	72%	14.92%
Kinect	79%	88%	8.71%
OpenPose	69%	75%	11.21%
HRNet	74%	79%	9.67%
ours	82%	90%	7.94%

**Table 6:** Results of Mediapipe and our method for detecting the critical point of the right knee during gait balance training.

Method	Joint Accuracy (Threshold = 8)	Difference Percentage
Mediapipe	65%	13.54%
ours	88%	7.35%

**Table 7:** Results of Mediapipe and our method for detecting the critical point of the right knee during center of gravity shift training.

Method	Joint Accuracy (Threshold = 8)	Difference Percentage
Mediapipe	75%	12.86%
ours	91%	6.83%

**Table 8:** Indicators of various methods in gait balance function rehabilitation training.

Method	MSE	R^2^	Cosine Similarity	Joint Accuracy
Kinect 2.0	51.6	0.85	0.84	80%
Mediapipe	38.9	0.86	0.88	82%
OpenPose	33.5	0.88	0.92	85%
HRNet	27.6	0.91	0.87	87%
ours	14.4	0.96	0.92	89%

**Table 9:** Indicators of various methods in center of gravity shift rehabilitation training.

Method	MSE	R^2^	Cosine Similarity	Joint Accuracy
Kinect 2.0	71.1	0.84	0.83	77%
Mediapipe	59.2	0.91	0.86	79%
OpenPose	46.7	0.87	0.87	83%
HRNet	39.8	0.89	0.86	84%
ours	20.5	0.93	0.90	88%

**Table 10:** Results of statistical analysis of the coefficient of variation of the repeated sequences of the subjects.

	1	2	3	4	5	6	7	8	9	10	11	12	13
Gait balance	8.82	7.52	5.53	5.36	8.73	3.16	5.80	3.50	6.54	9.31	8.38	6.33	8.27
Gravity shift	6.09	4.74	6.70	3.21	5.96	5.71	9.61	9.00	9.81	5.69	7.33	7.78	5.62

**Table 11:** Results of mean square error ANOVA between the method of this paper and the two base algorithms.

	F	*p*
Gait balance	3165.7	$3.6\times 10^{-10}$
Gravity shift	4794.7	$2.1\times 10^{-9}$

**Table 12:** Simulated-light images and real-light images ssim and psnr metrics.

	SSIM	PSNR
Gait balance	0.81	30.84
Gravity shift	0.88	35.21

## Data Availability

The data presented in this study are available on request from the corresponding author.
